# Endothelial NOX5 Obliterates the Reno-Protective Effect of Nox4 Deletion by Promoting Renal Fibrosis via Activation of EMT and ROS-Sensitive Pathways in Diabetes

**DOI:** 10.3390/antiox13040396

**Published:** 2024-03-26

**Authors:** Karin A. M. Jandeleit-Dahm, Haritha R. Kankanamalage, Aozhi Dai, Jaroslawna Meister, Sara Lopez-Trevino, Mark E. Cooper, Rhian M. Touyz, Christopher R. J. Kennedy, Jay C. Jha

**Affiliations:** 1 Department of Diabetes, School of Translational Medicine, Monash University, Alfred Medical Research & Education Precinct, Melbourne, VIC 3004, Australia; karin.jandeleit-dahm@monash.edu (K.A.M.J.-D.); lope0003@student.monash.edu (S.L.-T.); mark.cooper@monash.edu (M.E.C.); 2 Institute for Clinical Diabetology, German Diabetes Centre, Leibniz Centre for Diabetes Research at Heinrich Heine University, 40225 Düsseldorf, Germany; jaroslawna.meister@ddz.de; 3 Research Institute of the McGill University Health Centre, McGill University, Montreal, QC H3H 2R9, Canada; rhian.touyz@mcgill.ca; 4 Department of Medicine, Kidney Research Centre, Ottawa Hospital Research Institute, Ottawa, ON K1Y 4E9, Canada; ckennedy@uottawa.ca

**Keywords:** reactive oxygen species (ROS), NOX4, NOX5, diabetic kidney disease, fibrosis, inflammation

## Abstract

Chronic hyperglycemia induces intrarenal oxidative stress due to the excessive production of reactive oxygen species (ROS), leading to a cascade of events that contribute to the development and progression of diabetic kidney disease (DKD). NOX5, a pro-oxidant NADPH oxidase isoform, has been identified as a significant contributor to renal ROS in humans. Elevated levels of renal ROS contribute to endothelial cell dysfunction and associated inflammation, causing increased endothelial permeability, which can disrupt the renal ecosystem, leading to progressive albuminuria and renal fibrosis in DKD. This study specifically examines the contribution of endothelial cell-specific human *NOX5* expression in renal pathology in a transgenic mouse model of DKD. This study additionally compares NOX5 with the previously characterized NADPH oxidase, NOX4, in terms of their relative roles in DKD. Regardless of NOX4 pathway, this study found that endothelial cell-specific expression of *NOX5* exacerbates renal injury, albuminuria and fibrosis. This is attributed to the activation of the endothelial mesenchymal transition (EMT) pathway via enhanced ROS formation and the modulation of redox-sensitive factors. These findings underscore the potential therapeutic significance of NOX5 inhibition in human DKD. The study proposes that inhibiting NOX5 could be a promising approach for mitigating the progression of DKD and strengthens the case for the development of NOX5-specific inhibitors as a potential therapeutic intervention.

## 1. Introduction

Diabetic kidney disease (DKD) is recognized as the leading cause of end-stage renal failure worldwide, posing a major global health burden [1,2,3]. Despite the availability of current therapeutic approaches, including glucose-lowering agents, such as sodium-glucose cotransporter 2 (SGLT2) inhibitors and glucagon-like peptide-1 (GLP-1) agonists as well as renin–angiotensin–aldosterone system (RAAS) blockers, there is no cure yet, and a significant number of diabetic subjects progress to end-stage renal disease (ESRD) despite the use of current treatments [4,5,6,7,8]. Both clinical and experimental studies strongly implicate enhanced levels of intrarenal reactive oxygen species (ROS) leading to oxidative stress as a major determinant in the pathogenesis of DKD [9,10,11,12,13,14,15]. The most widely studied NADPH-oxidase isoform, NOX4, has been shown to be a key contributor to renal ROS generation, at least in murine (mouse and rat) models of DKD [13,14,15,16,17]. However, in humans, we and others have recently identified the potentially more important NADPH-oxidase isoform, NOX5, as a major player in DKD [18,19,20,21,22], and potentially in other complications of diabetes, including cardiovascular disease (CVD) [23,24] and retinopathy [25]. NOX5 is expressed in both glomerular [19,20,22] and tubular cells [26], and it primarily generates superoxide (O_2_^−^). Increased renal expression of NOX5 has been shown in diabetic individuals with nephropathy [19,20] as well as in response to high glucose in key renal cell populations implicated in DKD, such as proximal tubular cells, podocytes, mesangial, and endothelial cells [20,21,22,26]. In addition, the silencing of *NOX5* showed attenuation of the high-glucose-induced upregulation of markers of inflammation and fibrosis via a reduction in ROS formation in human mesangial cells [20,21]. Unlike other NOX isoforms, NOX5 activation does not require NADPH oxidase subunits, but it involves numerous regulatory processes, including changes in intracellular calcium, phosphorylation, and interaction with regulatory proteins, such as protein kinase C (PKC) [27,28,29,30]. Since murine species lack, but humans harbor NOX5 in their genome, various cell-specific *NOX5* transgenic mice models have been developed to define the role of NOX5 in DKD [18,20,22]. Recently, we and others have reported that the overexpression of human *NOX5* in podocytes and smooth muscle cells (mesangial cells) promotes renal damage in diabetes [18,19,20,22]. Considering the view that both NOX4 and NOX5 appear to play roles in DKD, we examined the pathogenic role of NOX5 in the presence or absence of NOX4 expression using endothelial cell (EC)-specific *NOX5* transgenic mice in a streptozotocin (STZ)-induced insulin-deficient type 1 diabetes model. The main focus of this study was to identify whether *NOX5* overexpression in endothelial cells (EC-*NOX5*), a key component of the glomerular filtration barrier, induces renal pathology, particularly renal inflammation and EMT-related fibrosis in the presence or absence of diabetes. Since NOX4 and NOX5 are both endogenously expressed in humans, we examined if Nox4 deletion is sufficient to protect against kidney damage in diabetes in the presence of *NOX5* expression.

## 2. Materials and Methods

### 2.1. Experimental Design

Genetically modified male *NOX5* transgenic mice with and without NOX4 expression in the presence or absence of diabetes were used for this study. The studies were approved by the Alfred Medical Research & Education Precinct (AMREP) Animal Ethics Committee under the guidelines provided by the National Health and Medical Research Council of Australia (ethics number E/1493/2014/B). Based on the results of our previous studies, 15 mice per group were randomly assigned to the respective animal study groups [16,20]. *NOX5* transgenic mice on an FVB/N background with a selective expression of the human *NOX5* gene in their endothelial cells (ECs) were generated as described previously [22]. Briefly, to generate EC-specific *NOX5* transgenic mice, the VEcad-tTA-FVB/N strain (VEcad: encoded by the *VEcad* promoter, also known as *Cdh5)* was crossed with the *NOX5 β* FVB/N strain (Clontech, Mountain View, CA, USA) to produce *VEcad*^+^*NOX5*^+^ and *VEcad*^+^*NOX5^−^* mice, respectively. A subgroup of *VEcad*^+^*NOX5*^+^ mice was crossed with in house-established *Nox4^−/−^* mice [16,31] to generate *VEcad*^+^*NOX5*^+^/*Nox4^−/−^* and *VEcad*^+^*NOX5*^−^/*Nox4^−/−^* mice, respectively. NOX5 protein expression in the endothelial cells was confirmed via the co-localization of NOX5 and CD31 (a marker of endothelial cells) (Supplementary Figure S1) in the glomeruli of *VEcad^+^NOX5^+^* transgenic mice. Diabetes was induced in 6-week-old male mice via five daily intraperitoneal injections of streptozotocin (Sigma-Aldrich, St. Louis, MO, USA) at a dose of 55 mg/kg in citrate buffer, with the control mice receiving citrate buffer alone. The mice were followed for 10 weeks.

### 2.2. Assessment of Metabolic Parameters and Renal Function

Urine samples were collected by individually placing mice into metabolic cages (Iffa Credo, Lyon, France) for 24 h during week 10 of the study. The albumin concentration in the urine was measured by using a mouse albumin ELISA quantification kit (Bethyl Laboratories, Montgomery, TX, USA). Blood glucose, glycated haemoglobin, and systolic blood pressure were measured, as described previously [16,20]. A mouse cystatin C ELISA kit (BioVendor, Brno, Czech Republic) was used to measure plasma cystatin C levels according to the manufacturer’s instructions. Mice were anesthetized via i.p. injection of sodium pentobarbital (100 mg/kg body weight; Euthatal; Sigma-Aldrich, Castle Hill, Australia) after 10 weeks (at 16 weeks of age). Both right and left kidneys were subsequently dissected, weighed and fixed in 10% formalin and embedded in paraffin as well as fresh frozen in liquid nitrogen for storage at −80 °C. Only diabetic mice with a blood glucose of ≥15 mmol/l were included in the experiments; mice with a blood glucose of <15 mmol/l and with polycystic kidneys were excluded from the study (<5% of the total number of mice).

### 2.3. Quantitative RT-PCR

The Trizol method was used for the extraction of renal cortical total RNA, and cDNA was constructed as described previously [16,20]. Respective mouse probes and primers were used for gene expression analysis, as described in Supplementary Table S1 using the Taqman system (ABI Prism 7500; PerkinElmer, Foster City, CA, USA). The relative mRNA expression was determined by normalizing the fluorescence signals to housekeeping gene 18S (18S ribosomal RNA Taqman Control Reagent kit) using the 2^−ΔΔCT^ fold change method. The results were expressed relative to the respective non-diabetic control mice without *NOX5* expression, which were arbitrarily assigned a value of 1.

### 2.4. Renal Histology and Immunohistochemistry

Three-micrometer-thick paraffin kidney sections were stained with periodic acid–Schiff in order to assess the mesangial area, glomerulosclerotic index (GSI), and tubulointerstitial injury (TII), as described previously [16,20]. Immunostaining for glomerular nitrotyrosine (rabbit anti-nitrotyrosine, catalog no. ab5411; Millipore, Billerica, MA, USA), collagen IV (goat anti-type IV collagen, catalog no. 1340-01; Southern Biotech, Birmingham, AL, USA), F4/80 (rat monoclonal anti-F4/80, catalog no. ab16911, Abcam, Cambridge, UK, CD68 (rabbit polyclonal anti-CD68, catalog no. ab125212; LSBio, Seattle, WA, USA), and alpha smooth muscle Actin (rabbit polyclonal anti-α-SMA, catalog no. ab5694, Abcam, Cambridge, UK) were performed as described previously [16,20,21,22]. The sections were then examined under a light microscope (Olympus BX-50; Olympus Optical, Tokyo, Japan) with 20 glomeruli being captured per kidney for the assessment of mesangial area and the quantification of nitrotyrosine, collagen IV, α-SMA, and F4/80 (percentage of glomerular area) using Image-Pro plus 7.0 software (Media Cybernetics, Rockville, MD, USA). GSI and TII were graded, as described previously [16,20,21,22]. The number of CD68 positively stained macrophages within each glomerulus was counted. All of the assessments were performed in a blinded manner. The results were expressed relative to the respective non-diabetic control mice without EC-*NOX5* expression, which were arbitrarily assigned a value of 1.

### 2.5. Protein Expression of Renal MCP-1 by ELISA

The renal MCP-1 level was determined by using the Quantikine Mouse MCP-1 ELISA kit (R&D Systems, Minneapolis, MN, USA), as per the kit’s instructions. The renal MCP-1 level was expressed relative to the total protein concentration [16].

### 2.6. Renal Protein Expression via Western Blot

The renal protein expressions of protein kinase C alpha (PKC-α) and nuclear factor erythroid 2-related factor 2 (NRF2) were determined via Western blot analysis, as described previously [20,22]. Briefly, protein extracts from the renal cortex were electrophoresed on 4–20% Mini-PROTEAN precast gels (Bio-Rad Laboratories, Richmond, CA, USA) under non-reducing conditions. The blots were incubated with PKC-α (rabbit monoclonal; catalog no. ab32376, Abcam) and NRF2 (goat polyclonal, catalog no. SAB2501713; Sigma-Aldrich-Merck) overnight at 4 °C, followed by incubation with goat anti-rabbit or rabbit anti-goat secondary antibodies, respectively (Dako Corp., Carpinteria, CA, USA). Membranes were subsequently probed for β-actin (Sigma-Aldrich) to confirm equal loading of samples. An ECL detection kit (Sigma-Aldrich) was used for the detection of blots and quantified via densitometry using Quantity-One software 4.6 (Bio-Rad Laboratories).

### 2.7. Statistical Analysis

All of the variables were analysed via two-way ANOVA using GraphPad Prism 7 (San Diego, CA, USA) for multiple comparison of the means followed by Tukey’s post hoc test or analysed using a two-tailed unpaired t-test where required. A *p* value of < 0.05 was considered to be statistically significant. ‘ns’ indicates not significant. The results are expressed as mean ± SEM unless otherwise specified.

## 3. Results

### 3.1. Metabolic Variables and Renal Function

In order to examine the pathogenic role of EC-*NOX5* expression in DKD independent of the NOX4 pathway, the study was conducted in both wild-type (WT) and Nox4 knockout (KO) mice with and without EC-*NOX5* expression in the STZ mouse model of diabetes. In the Nox4WT study group (Table 1), diabetic mice with (*VEcad^+^Nox5^+^/Nox4*WT) or without (*VEcad^+^Nox5^−^/Nox4*WT) EC-*NOX5* expression had elevated levels of plasma glucose and glycated hemoglobin, reduced body weights, and increased kidney weight/body weight ratios but unchanged systolic blood pressure when compared to the non-diabetic control groups (Table 1). The induction of diabetes with EC-*NOX5* expression had no significant effect on these metabolic variables (Table 1). Similarly, the Nox4KO group (Table 2) showed no significant changes in metabolic variables between diabetic mice with (*VEcad^+^Nox5^+^/Nox4^−/−^)* and without (*VEcad^+^Nox5^−^/Nox4^−/−^)* EC-*NOX5* expression (Table 2). However, systolic blood pressure was higher in *VEcad^+^Nox5^+^/Nox4^−/−^* diabetic mice compared to the non-diabetic control mice (Table 2). To examine renal function, we assessed plasma cystatin C levels. In comparison to the respective non-diabetic control mice, all groups of diabetic mice with and without EC-*NOX5* expression showed lower levels of plasma cystatin C, indicating a state of hyperfiltration, as seen in the early stages of DKD (Table 1 and Table 2). EC-*NOX5* expression per se did not alter the state of hyperfiltration. However, relative to WT mice, Nox4KO mice with and without EC-*NOX5* expression had lower cystatin C levels (Table 1 and Table 2).

### 3.2. EC-NOX5 Increases Albuminuria and Renal Injury in WT and Nox4KO Mice in Diabetes

**Figure 1:**
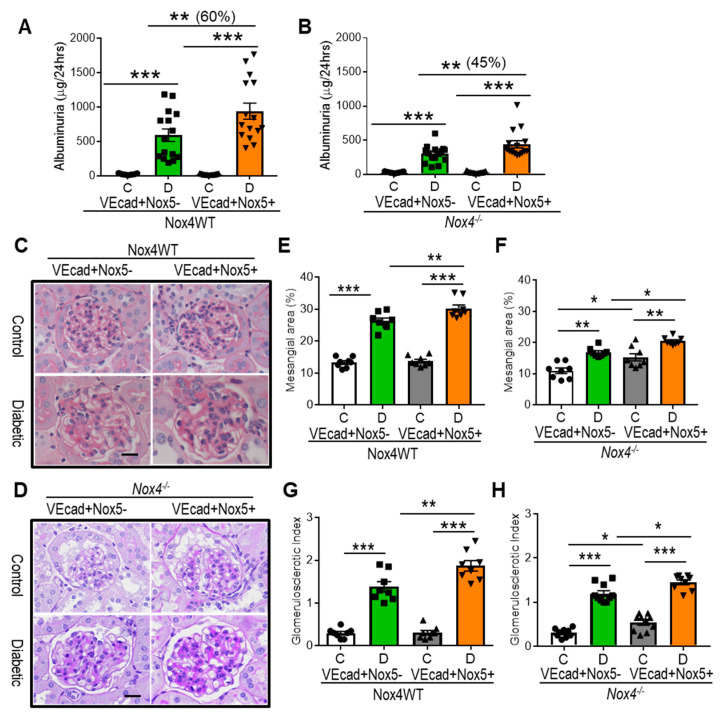
EC*NOX5* increases albuminuria and renal injury in diabetes. Assessment of albuminuria (n = 15/gp) (**A**,**B**), periodic acid-Schiff staining (**C**,**D**), relative mesangial area expansion (**E**,**F**) and glomerulosclerotic index (**G**,**H**) in Nox4 wild-type (WT) and Nox4 knockout (*Nox4^−/−^)* mice with and without *NOX5* expression after 10 weeks of STZ diabetes. Scale bar: 20 μm, in all photomicrographs (n = 7–10/gp). Data are shown as mean ± SEM. Asterisks represent *p*-values for comparisons of the indicated groups: * <0.05, ** <0.01 and *** <0.001.

Regardless of NOX4 expression, increased levels of albuminuria (*p* < 0.001) were found in all groups of mice after 10 weeks of STZ-diabetes when compared to the respective control groups (Figure 1A,B). In the absence of EC-*NOX5* expression, Nox4 knockout (293 ± 37 µg/24 h, Figure 1B) diabetic mice had lower levels of albuminuria in comparison to WT (593 ± 102 µg/24 h, Figure 1A) diabetic mice. Importantly, the EC-specific overexpression of human *NOX5* led to a further increase in albuminuria, both in the WT (60%, 942 ± 131 µg/24 h, Figure 1A) and Nox4 knockout (45%, 442 ± 56 µg/24 h; Figure 1B) diabetic mice. In non-diabetic mice, EC-*NOX5* expression did not affect albuminuria when compared to mice without *NOX5* expression (Figure 1A,B). In addition to albuminuria, we examined mesangial expansion, glomerulosclerosis (GSI), and tubulointerstitial injury (TII) in order to assess renal structural damage in diabetes. The induction of diabetes increased mesangial expansion and glomerulosclerosis, which were further increased by EC-*NOX5* expression in WT mice (Figure 1C,E,G). In addition, despite Nox4 deletion, both mesangial expansion and GSI remained increased in diabetic mice in comparison to their non-diabetic counterparts, with EC-*NOX5* expression further exacerbating these renal structural parameters (Figure 1D,F,H). Notably, non-diabetic mice with EC-*NOX5* expression showed a significant increase in mesangial area and GSI (*p* < 0.05) in Nox4 knockout mice (Figure 1D,F,H). Unlike glomerular injury, no change in TII was observed in all groups of mice in the presence or absence of diabetes (Supplementary Figure S2A–D).

### 3.3. EC-NOX5 Enhances Renal ROS Formation in WT and Nox4KO Mice in Diabetes

**Figure 2:**
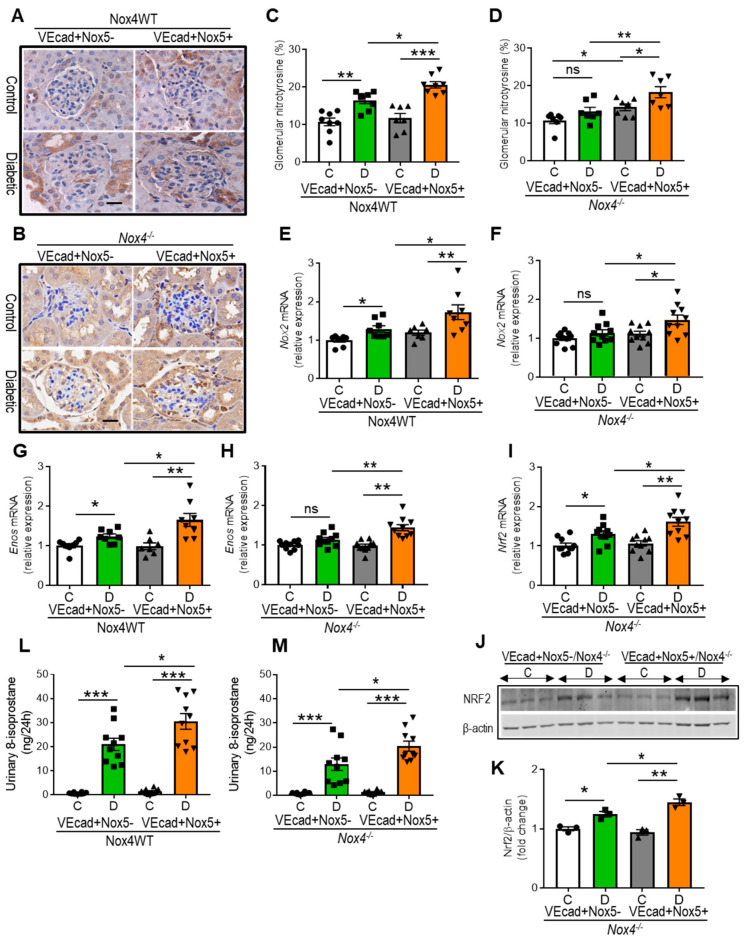
EC-*NOX5* enhances renal ROS formation in diabetes. Immunostaining of glomerular nitrotyrosine (**A**,**B**) and its quantification (**C**,**D**), gene expression of renal *Nox2* (**E**,**F**) and *eNOS* (**G,H**) and urinary 8-isoprostane levels (**L**,**M**) in Nox4WT and Nox4^−/−^ mice with and without *NOX5* expression after 10 weeks of STZ-induced diabetes (n = 7–10/gp). Scale bar: 20 μm, in all photomicrographs. Gene and protein expression of NRF2 in Nox4^−/−^ mice with and without *NOX5* expression after 10 weeks of STZ-induced diabetes (**I**–**K**). Western blot showing the protein expression of renal NRF2 (61 kDa) and its quantification (**J**,**K**) (n = 3/group). β-Actin (42 kDa) serves as a housekeeping protein. Data are shown as mean ± SEM. Asterisks represent *p*-values for comparisons of the indicated groups: * <0.05, ** <0.01, *** <0.001 and ns, not significant.

EC-*NOX5* overexpression increased the accumulation of glomerular nitrotyrosine, a marker of oxidative stress, in diabetic WT mice, suggestive of further-enhanced ROS formation in the presence of EC-*NOX5* (Figure 2A,C). In addition, the induction of diabetes increased the expression of renal NADPH-oxidase-2 (*Nox2*) and endothelial nitric oxide synthase (*eNOS*), which were further amplified by EC-*NOX5* expression (Figure 2E,G). However, the increased renal expression of NADPH-oxidase-4 (*Nox4*) was downregulated by EC-*NOX5* expression in cases of diabetes (Supplementary Figure S2E). In contrast, in the Nox4KO mice, no differences were observed in the glomerular accumulation of nitrotyrosine or the gene expression of *Nox2* and *eNOS* in diabetes compared to the controls (Figure 2B,D,F,H). Adversely, diabetic EC-*NOX5*-expressing mice that lack NOX4 showed an increased accumulation of glomerular nitrotyrosine and the upregulation of both *NOX2* and *eNOS* compared to respective non-diabetic and diabetic mice without *NOX5* expression (Figure 2B,D,F,H). Notably, EC-*NOX5*-expressing non-diabetic mice had more glomerular nitrotyrosine compared to the control mice without *NOX5* expression (Figure 2B,D). Additionally, in the Nox4 knockout group, we examined the expression of a transcription factor, NRF2, which is shown to be associated with antioxidant-redox signaling. The renal expression of NRF2 was increased by diabetes at both gene and protein levels, with EC-*NOX5* expression further increasing the level of NRF2 expression (Figure 2I–K). We also examined urinary 8-isoprostane as a marker of systemic oxidative stress. All groups of diabetic mice showed elevated levels of urinary 8-isoprostane when compared to their respective non-diabetic counterparts (Figure 2L,M). In addition, EC-*NOX5* expression showed a further increase in urinary 8-isoprostane excretion in both WT and Nox4KO diabetic mice compared to mice without *NOX5* expression (Figure 2L,M).

### 3.4. EC-NOX5 Upregulates Markers of Inflammation and ROS-Sensitive Factors in WT and Nox4KO Mice in Diabetes

**Figure 3:**
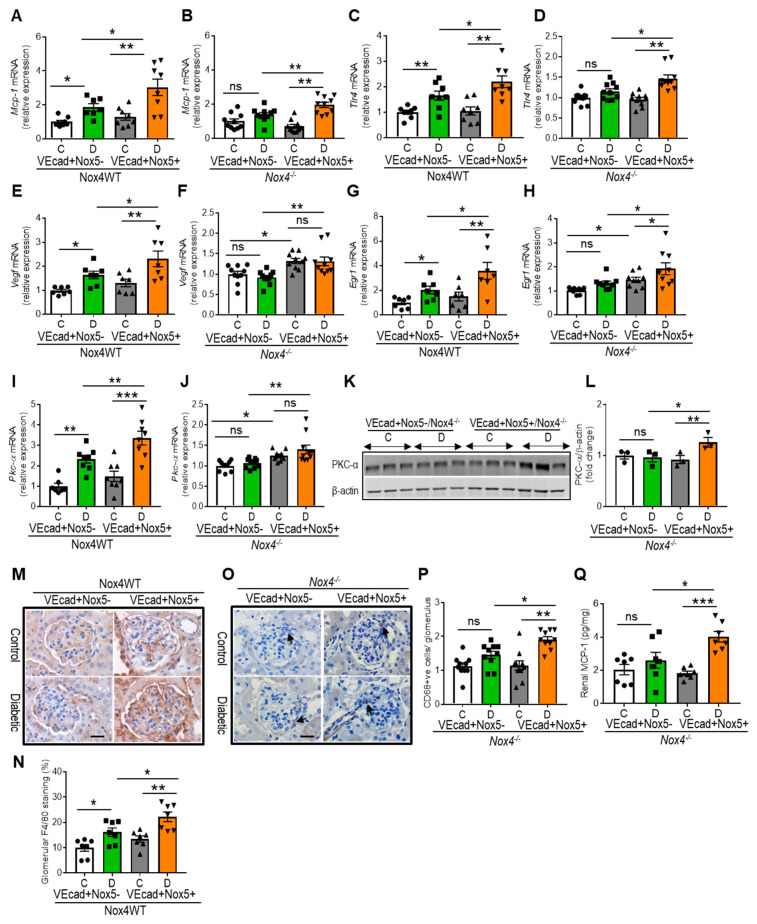
EC-*NOX5* upregulates markers of inflammation and ROS-sensitive factors. Gene expression of renal *Mcp-1* (**A**,**B**), *Tlr4* (**C**,**D**), *Vegf* (**E**,**F**), *Egr1* (**G**,**H**), and *Pkc-α* (**I**,**J**) in Nox4WT and Nox4^−/−^ mice with and without *NOX5* expression after 10 weeks of STZ-induced diabetes (n = 7–10/gp). Western blot showing the protein expression of renal PKC-α (77 kDa) and its quantification (**K**,**L**) (n = 3/group). β-Actin (42 kDa) serves as a housekeeping protein. Immunostaining for F4/80 (**M**) and CD68 (**O**) and their quantification (**N**,**P**) as well as protein level of MCP-1 (**Q**) by ELISA (n = 7–10/gp). Scale bar: 20 μm, in all photomicrographs. Data are shown as mean ± SEM. Asterisks represent *p*-values for comparisons of the indicated groups: * <0.05, ** <0.01, *** <0.001 and ns, not significant.

We examined the renal expression of pro-inflammatory cytokines, including monocyte chemoattractant protein-1 (MCP-1), Toll-like receptor-4 (TLR4), and a vascular endothelial growth factor, VEGF (Figure 3A–F), as well as ROS-sensitive factors, such as a transcription factor, EGR1 and protein kinase-α, PKC-α (Figure 3G–L). Diabetic mice showed increased expression of *Mcp-1*, *Tlr4*, *Vegf*, *Pkc-α*, and *Egr1* in the Nox4 wild-type study group, with the further upregulation of these genes in the presence of EC-*NOX5* expression (Figure 3A,C,E,G,I). In the Nox4KO group, the expression of these genes remained unchanged in diabetic mice in the absence of *NOX5* expression when compared to their respective non-diabetic control mice (Figure 3B,D,F,H,J). By contrast, all of these factors were increased by EC-*NOX5* expression in cases of diabetes. Moreover, non-diabetic mice with EC-*NOX5* expression showed the upregulation of *Vegf*, *Pkc-α*, and *Egr1* compared to the control mice without *NOX5* expression (*p*< 0.05, Figure 3F,H,J). Additionally, the protein expression of PKC-α was increased in diabetic mice expressing EC-*NOX5* (Figure 3K,L). We also examined the macrophage markers F4/80 (Figure 3M,N) and CD68 (Figure 3O,P) using immunohistochemistry, and the MCP-1 protein level using ELISA (Figure 3Q). Similar to the aforementioned pro-inflammatory cytokines, EC-*NOX5* expression in the wild-type diabetic mice showed a further increase in glomerular F4/80 expression compared to mice without *NOX5* expression (Figure 3M,N). Furthermore, in the Nox4KO group, no change in glomerular CD68-positive cells and renal MCP-1 level was found in the diabetic mice when compared to the non-diabetic control mice (Figure 3O–Q). However, in the presence of EC-*NOX5* expression, there was an increased number of glomerular CD68-positive cells and increased renal MCP-1 levels in cases of diabetes (Figure 3O–Q).

### 3.5. EC-NOX5 Upsurges Renal ECM Accumulation and Fibrosis in WT and Nox4KO Mice

**Figure 4:**
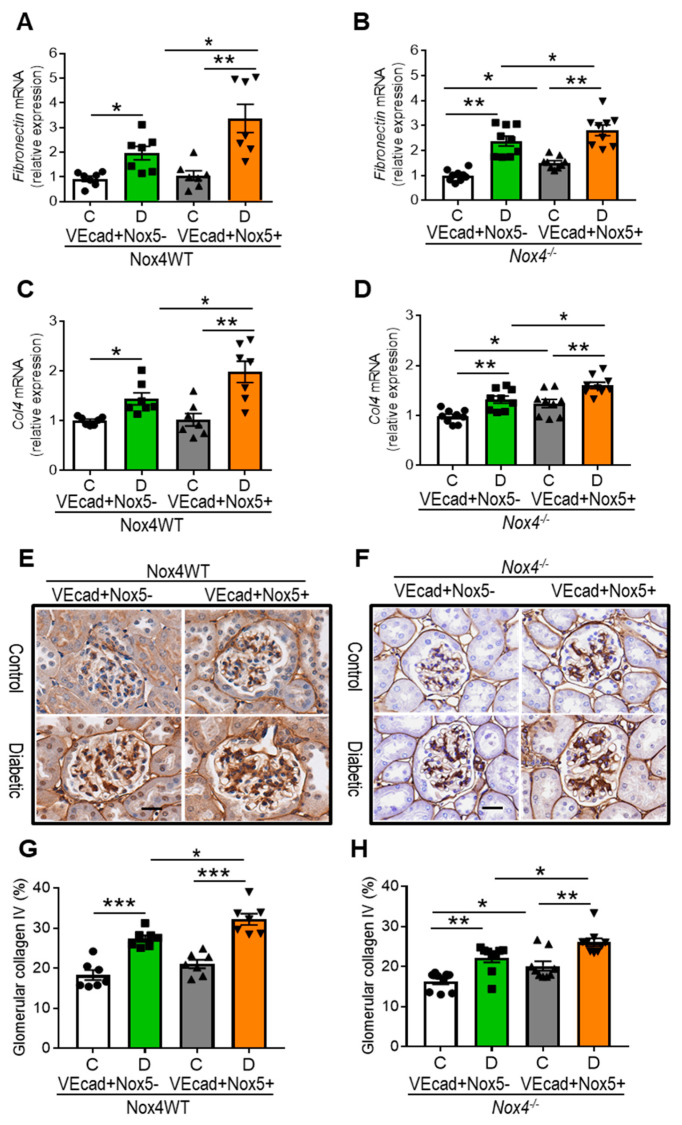
EC-*NOX5* upsurges ECM accumulation and renal fibrosis. The renal gene expression of fibronectin (*Fn1*) (**A**,**B**) and collagen IV (*Col4*) (**C**,**D**) as well as immunostaining of collagen IV (**E**,**F**) and its quantification (**G**,**H**) in Nox4WT and Nox4^−/−^ mice with and without *NOX5* expression after 10 weeks of STZ-induced diabetes. Scale bar, 20 μm in all photomicrographs (n = 7–10/gp). Data are shown as mean ± SEM. Asterisks represent *p*-values for comparisons of the indicated groups: * <0.05, ** <0.01 and *** <0.001.

The accumulation of extracellular matrix (ECM) is an important feature of renal fibrosis in DKD [15]. In this study, we examined the expression of fibronectin and collagen IV as classical components of the ECM. Consistent with increased mesangial expansion and glomerulosclerosis, EC-*NOX5* expression in WT diabetic mice further increased the gene expression of fibronectin and collagen IV (Figure 4A,C) and enhanced the accumulation of collagen IV at the protein level (Figure 4E,G). By contrast, despite Nox4 deletion (Figure 4B,D,F,H), diabetic mice showed renal upregulation of these ECM components in comparison to their respective non-diabetic counterparts, with these components further upregulated by EC-*NOX5* expression and diabetes. Moreover, non-diabetic mice with Nox4 deletion showed increased expression of both collagen IV and fibronectin when expressing *NOX5* in EC compared to their respective control mice without *NOX5* expression (Figure 4B,D,F,H).

### 3.6. EC-NOX5 Promotes Renal Fibrosis by Activating EMT-Related Factors

**Figure 5:**
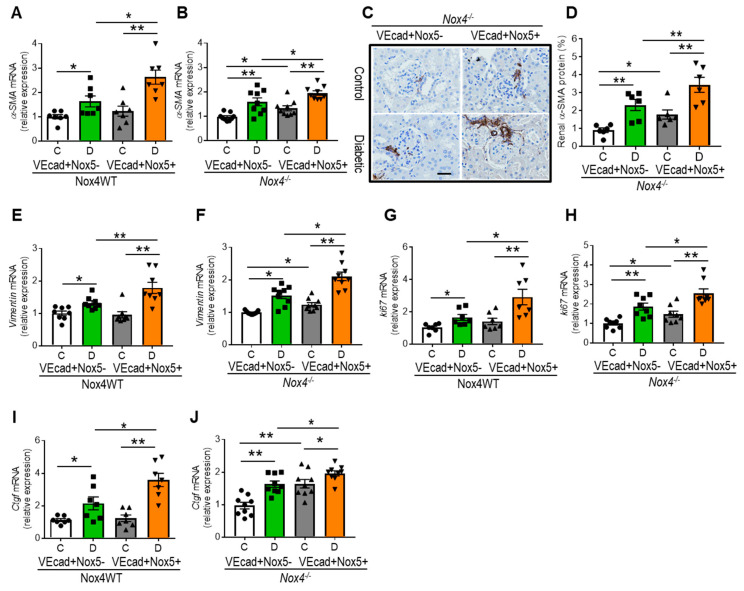
EC-*NOX5* promotes renal fibrosis by activating EMT-related factors. Gene expression of renal *α-SMA* (**A**,**B**), *Vimentin* (**E**,**F**), *Ki67* (**G**,**H**), and *Ctgf* (**I**,**J**) in Nox4WT and Nox4^−/−^ mice with and without *NOX5* expression after 10 weeks of STZ-induced diabetes (n = 7–10/gp). Immunostaining of renal α-SMA (**C**) and its quantification (**D**) (n = 6/gp). Scale bar: 20 μm, in all photomicrographs. Data are shown as mean ± SEM. Asterisks represent *p*-values for comparisons of the indicated groups: * <0.05 and ** <0.01.

Epithelial–mesenchymal transition (EMT) is a key pathological process in renal fibrosis, with a pro-oxidant environment activating EMT in chronic diseases, including DKD. We examined EMT markers such as α-smooth muscle actin (*α-SMA*) and *vimentin* as well as EMT-related factors, such as a cell proliferation marker, *Ki67*, and a prosclerotic factor, connective tissue growth factor, *Ctgf* (Figure 5A–J). Similar to the enhanced renal upregulation of collagen IV and fibronectin, all of these EMT markers and related factors were increased by diabetes in the WT mice, with a further increase as a result of EC-*NOX5* expression in diabetes (Figure 5A,E,G,I). In contrast, despite Nox4 deletion, diabetic mice showed increased expression of *α-SMA*, *vimentin*, *Ki67*, and *Ctgf* in comparison to their respective non-diabetic counterparts, with these genes further upregulated by EC-*NOX5* expression in diabetic mice (Figure 5B,F,H,J). Moreover, similar to gene expression, the renal protein expression of α-SMA was also further increased by EC-*NOX5* expression and diabetes (Figure 5C,D). Notably, all of these EMT-related factors were significantly upregulated in the presence of EC-*NOX5* expression, even in the non-diabetic condition (Figure 5B–D,F,H,J).

## 4. Discussion

Intrarenal oxidative stress driven by the overproduction of ROS plays a central role in the pathogenesis of DKD. Numerous experimental studies, including our own, have shown the deleterious role of the constitutively active NADPH oxidase isoform, NOX4, also known as Renox, in DKD [13,14,15,16,17,32]. Indeed, targeting NOX4 via genetic deletion or pharmacological inhibition afforded renoprotection, as reflected by the attenuation of diabetes-induced enhanced albuminuria, renal inflammation, and fibrosis via a reduction in ROS formation [13,16,17]. However, a clinical trial outcome in patients with type 2 diabetes and kidney disease by Genkyotex using a dual NOX1/4 inhibitor, GKT137831, was negative as the inhibitor was unable to reduce albuminuria, which was the primary endpoint [33]. Additionally, this compound is currently under evaluation in adults with type 1 diabetes and kidney disease [34]. On the other hand, NOX4 appears to have a vasculoprotective role, as the genetic deletion of Nox4 in mice led to accelerated atherosclerosis in diabetes [35]. Since individuals with diabetes develop both renal and vascular complications simultaneously, targeting solely NOX4 systemically is neither desirable nor recommended.

In the quest for a superior yet feasible target for the treatment of diabetic complications, recently, more consideration has been given to the NOX5 isoform, which is present in humans but absent in rodents and is highly expressed by both renal and vascular cells in hyperglycemic states [19,20,21,23,29,36,37]. Recent studies, including our own, have clearly demonstrated the increased expression of renal NOX5 in individuals with diabetes [19,20,21]. In addition, *NOX5*-transgenic mouse models with cell-specific expression of human *NOX5*, particularly in the podocytes and mesangial cells, demonstrated the pathogenic role of NOX5 in DKD [19,20,21]. Expanding on previous findings, this study examined the role of endothelial cell-specific *NOX5* expression in an STZ-induced insulin-deficient diabetes mouse model in the presence or absence of NOX4 expression. It was demonstrated that EC-*NOX5* expression further enhanced diabetes-induced increased albuminuria, inflammation, and fibrosis, as well as the upregulation of ROS-sensitive factors, regardless of NOX4 expression. These findings highlight the dominant role of NOX5 over NOX4 in the progression of DKD.

NOX5 primarily generates superoxide, which interacts with nitric oxide (NO) to form peroxynitrite, which in turn binds to tyrosine residues to produce nitrotyrosine, a marker of ROS [15,20]. A low level of nitric oxide (NO) bioavailability is evident in endothelial dysfunction and chronic kidney disease [38]. In this study, enhanced levels of glomerular nitrotyrosine and the upregulation of endothelial NO synthase (eNOS) by EC-*NOX5* expression suggest that superoxide derived from EC-*NOX5* potentially utilizes endothelial NO to form nitrotyrosine, leading to the diminished bioavailability of NO, thus causing endothelial dysfunction followed by the promotion of renal injury in diabetes. Moreover, increased glomerular nitrotyrosine via EC-*NOX5* expression in Nox4KO mice highlights the predominant role of NOX5 over NOX4 in enhancing ROS formation. This also suggests that Nox4 deletion alone is insufficient to halt ROS formation in the presence of NOX5. NRF2 plays an important role in antioxidant defense mechanisms in various chronic diseases, including DKD [22,39,40,41]. The increased expression of NRF2 in diabetic mice with EC-*NOX5* expression suggests a potential compensatory effect in response to high levels of ROS formation due to the diabetic insult and NOX5 expression. Impairment in glomerular endothelial function likely contributes to albuminuria and DKD progression [42,43]. The development of albuminuria in diabetes is a result of pathological damage to the highly regulated glomerular filtration barrier, including ultrastructural injury to key renal cell populations such as endothelial cells and podocytes [15,43,44,45,46]. Previously, it has been shown in murine models of diabetes that the blockade of NOX4 leads to a reduction in albuminuria [14,15,16,17] and that the overexpression of human *NOX5* in podocytes and mesangial cells of diabetic mice exacerbates albuminuria [19,20]. In this study, EC-*NOX5* expression in both WT and Nox4KO diabetic mice increased the level of albuminuria by almost 50% in comparison to mice without *NOX5* expression. This is especially relevant in the context of human DKD, where both NOX4 and NOX5 are endogenously expressed and, as per these findings, inhibiting NOX4 would not sufficiently attenuate albuminuria in the presence of NOX5 expression. Moreover, this study explored the expression of VEGF, which was previously associated with endothelial dysfunction and albuminuria, and more recently linked with NOX5 in the context of diabetic retinopathy [21,22,25]. Previous findings also show a correlation between the renal expression of PKC-α, VEGF, and albuminuria, where PKC-α knockout diabetic mice had decreased renal VEGF expression in association with reduced albuminuria [47,48,49]. Supporting these previous findings, the current study demonstrated a further increase in both PKC-α and VEGF expression in EC-*NOX5*-expressing diabetic mice as opposed to mice without *NOX5* expression regardless of the status of NOX4 expression. These findings demonstrate a role for NOX5 in modulating both VEGF and PKC-α expression independent of NOX4 in the kidney, which may explain the pathway responsible for exacerbating albuminuria in response to NOX5 expression.

Renal inflammation in diabetes has been linked to NOX5, particularly via activation of the TLR-4-dependent pathway [18,21,22]. In the Akita mouse model of DKD, the overexpression of *NOX5* in smooth muscle cells (mesangial cells) showed the upregulation of TLR-4 and MCP-1 expression via the activation of the transcription factor NF-κB, which regulates cytokine production [18,21,22]. This study used a STZ mouse model of diabetes to examine the renal expression of TLR-4, a key regulator of inflammatory response and MCP-1, a chemokine that drives macrophage infiltration as well as F4/80 and CD68, key markers of inflammation, which are known to have augmented expression levels in experimental models of DKD [15,37,50,51,52]. We found the upregulation of these pro-inflammatory cytokines and increased glomerular macrophage infiltration as a result of EC-*NOX5* expression independent of the NOX4 pathway in STZ-induced DKD. This suggests that NOX5 is a key modulator in driving renal inflammation via the activation of these pro-inflammatory molecules in DKD.

Previous findings have identified an important role for ROS-sensitive factors, including PKC-α and EGR1, in driving renal inflammation and fibrosis in DKD [21,22,27,53,54,55,56,57]. PKC-α is known to have a role in directly influencing NOX5 activity via phosphorylation, and it has been previously demonstrated that activation of the PKC pathway is associated with enhanced renal oxidative stress and inflammation [21,22,27,58,59,60,61]. The transcription factor EGR1, which is expressed in several renal cell populations, has been shown to be associated with renal fibrosis and inflammation as well as being a transcriptional activator of NOX4 in DKD [55,56]. There is also evidence to suggest that both EGR1 and PKC-α are modulated by NOX5 since the silencing of *NOX5* in human mesangial cells led to the attenuation of high-glucose-induced increases in the expression of *Pkc-α* and *Egr1* [21]. Further validating previous findings, it is evident from this study that both PKC-α and EGR1 are modulated by both NOX5 and NOX4 in DKD since the genetic deletion of Nox4 attenuates and EC-*NOX5* expression augments these ROS-sensitive factors.

Oxidative stress is associated with the activation of pro-fibrotic pathways in the kidney, leading to the deposition of extracellular matrix proteins, tissue remodeling and, ultimately, renal sclerosis in DKD [9,11,15,62,63,64,65,66,67,68,69,70,71]. In order to examine the effect of EC-*NOX5* expression on pathological pathways causing renal injury, this study examined mesangial expansion, glomerulosclerosis, and markers of extracellular matrix genes and proteins, specifically collagen IV and fibronectin. Consistent with the finding of enhanced albuminuria, EC-*NOX5* expression exacerbated the aforementioned renal injury parameters in diabetic mice regardless of NOX4 expression. An important observation was seen in the Nox4KO study group, where EC-NOX5 expression showed increased mesangial expansion, glomerulosclerosis, and enhanced glomerular accumulation of collagen IV as well as the upregulation of collagen IV and fibronectin not only in the presence of diabetes but also in the absence of diabetes, suggesting a dominant direct role of NOX5 in promoting pro-fibrotic pathways. This is consistent with previous studies, where the silencing of *NOX5* in human mesangial cells but not *NOX4* led to the attenuation of high-glucose-induced upregulation of these markers of fibrosis, further highlighting the dominant role of NOX5 in exacerbating renal fibrosis in DKD [21]. Oxidative stress-mediated renal fibrosis involves cellular proliferation and differentiation as well as epithelial-to-mesenchymal (EMT) transition [15,72,73,74,75]. To further elucidate the link between oxidative stress and pro-fibrotic pathways, this study examined the influence of endothelial *NOX5* on markers of EMT, including smooth muscle actin-α (α-SMA) and vimentin, the intermediate filament protein recognized as an essential component regulating EMT as well as EMT-related factors such as the cell proliferation marker ki67 and the prosclerotic growth factor CTGF in DKD [11,15,73,76,77]. This study demonstrated that despite Nox4 deletion, EC-*NOX5* expression showed upregulation of these EMT markers and related pro-fibrotic factors in DKD. Importantly, this effect was also seen in non-diabetic mice lacking NOX4 expression, suggesting a more substantial role of NOX5 versus NOX4 in modulating EMT pathways leading to renal fibrosis.

## 5. Conclusions

The current study has provided extensive evidence that endothelial cell-specific *NOX5* expression further exacerbates renal injury in diabetes, as reflected by an increase in albuminuria, EMT-related factors, and renal fibrosis, as well as enhancing inflammation and activating ROS-sensitive pathways, even in the absence of NOX4 in the context of STZ-induced diabetes. Previous findings have demonstrated that the genetic deletion or pharmacological inhibition of NOX4 in diabetic murine models (without NOX5 expression) provides renoprotection by attenuating albuminuria as well as inflammatory and pro-fibrotic pathways via a reduction in ROS formation. However, in the present study, it was evident that the genetic deletion of NOX4 alone was insufficient to completely prevent diabetes-induced albuminuria, renal inflammation, and fibrosis, thus demonstrating the dominant role of NOX5 versus NOX4 in driving these pathological pathways in a model of DKD. This finding needs to be considered in the context of human DKD, where both NOX5 and NOX4 are endogenously expressed. In addition, the potential of targeting NOX5 therapeutically should be further explored in experimental models that more closely align with human DKD, such as in rabbits, which endogenously express both NOX4 and NOX5. This study’s findings also strengthen the rationale for the development of NOX5-specific inhibitors to combat DKD and thus alleviate its global health burden. In summary, this study contributes to our understanding of the molecular mechanisms underlying DKD, emphasizing the crucial role of NOX5 in promoting renal injury, fibrosis, and inflammation. The identification of NOX5 as a potential target provides a new avenue to develop therapies aimed at mitigating DKD.

## Figures and Tables

**Table 1:** Metabolic variables (n = 15/group) and plasma cystatin C level (n = 8/group) in control and diabetic *VEcad^+^Nox5^−^/Nox4*WT and *VEcad^+^Nox5^+^/Nox4*WT mice. Data are shown as mean ± SEM. * *p* < 0.05 and ** *p* < 0.01 vs. control *VEcad^+^Nox5^−^/Nox4*WT mice; ^^^ *p* < 0.05 and ^^^^ *p* < 0.01 vs. control *VEcad^+^Nox5^+^/Nox4*WT mice.

	*VEcad^+^Nox5^−^/Nox4*WT		*VEcad^+^Nox5^+^/Nox4*WT	
	Control	Diabetes	Control	Diabetes
Plasma glucose (mmol/L)	12.2 ± 0.6	33.1 ± 1.3 **	12.1 ± 0.4	33.8 ± 0.6 ^^^^
Glycated hemoglobin (%)	4.1 ± 0.1	10.3 ± 0.4 *	4.2 ± 0.1	10.2 ± 0.3 ^^^
Body weight (g)	31 ± 0.5	28 ± 0.9 *	30 ± 0.7	27 ± 0.5 ^^^
Kidney weight/Body weight (mg/g)	13.1 ± 0.3	21.1 ± 1.3 *	13.6 ± 0.5	23.1 ± 1.2 ^^^
Systolic BP (mmHg)	107 ± 1	111 ± 2	110 ± 2	109 ± 1
Plasma cystatin C (ng/mL)	450 ± 74	272 ± 36 *	428 ± 61	234 ± 27 ^^^

**Table 2:** Metabolic parameters (n = 15/group) and plasma cystatin C level (n= 8/group) in control and diabetic *VEcad^+^Nox5^−^/Nox4^−/−^* and *VEcad^+^Nox5^+^/Nox4^−/−^* mice. Data are shown as mean ± SEM. ^$^ *p* < 0.05 and ^$$^ *p* < 0.01 vs. control *VEcad^+^Nox5^−^/Nox4^−/−^* mice; ^#^ *p* < 0.05 and ^##^ *p* < 0.01 vs. control *VEcad^+^Nox5^+^/Nox4^−/−^* mice.

	*VEcad^+^Nox5^−^/Nox4^−/−^*		*VEcad^+^Nox5^+^/Nox4^−/−^*	
	Control	Diabetes	Control	Diabetes
Plasma glucose (mmol/L)	13.2 ±0.6	33.3 ± 0.1 ^$$^	12.7 ± 0.8	29.1 ± 1.7 ^##^
Glycated hemoglobin (%)	4.1 ± 0.1	11.1 ± 0.3 ^$^	4.1 ± 0.1	10.2 ± 0.6 ^#^
Body weight (g)	40 ± 1.2	32 ± 0.7 ^$^	36 ± 0.7	30 ± 0.9 ^#^
Kidney weight /Body weight (mg/g)	10.5 ± 0.3	18.4 ± 0.6 ^$^	11.6 ± 0.4	18.9 ± 0.9 ^#^
Systolic BP (mmHg)	120 ± 8	119 ± 7	110 ± 6	131 ± 8 ^#^
Plasma cystatin C (ng/mL)	417 ± 64	159 ± 16 ^$^	406 ± 41	177 ± 31 ^#^

## Data Availability

Data are contained within the article and Supplementary Materials. Additional data underlying this article will be shared on reasonable request to the corresponding author.

## Supplementary Materials

The following supporting information can be downloaded at: https://www.mdpi.com/article/10.3390/antiox13040396/s1, Figure S1: *NOX5* transgenic mice expressing human *NOX5* in endothelial cells (*VEcad^+^NOX5*^+^) in the glomerulus. Co-localization of CD31 (a marker of endothelial cells; green staining) and NOX5 (red staining) in frozen kidney sections of control VEcad^+^NOX5^+^ transgenic mouse. Scale bar, 20 μm in all photomicrographs; Figure S2: Periodic Acid-Schiff staining (A,B) and the scoring of tubulointerstitial injury, TII (C,D) in all groups of wild type and Nox4 knockout (Nox4^−/−^) mice with and without *NOX5* expression after 10 weeks of STZ-diabetes. Scale bar, 50 μm in all photomicrographs (n= 7–10/gp). Renal cortical gene expression of *Nox4* (E) in wild type mice with and without *NOX5* expression after 10 weeks of STZ-diabetes. Data are shown as mean ± SEM. Asterisks represent p-values for comparisons of the indicated groups: * <0.05; Table S1: Mouse probes and primers for RT-PCR.
