# Supplementary material for: Endothelial NOX5 Obliterates the Reno-Protective Effect of Nox4 Deletion by Promoting Renal Fibrosis via Activation of EMT and ROS-Sensitive Pathways in Diabetes
Source: Antioxidants (Basel). 2024 Mar 26;13(4):396. doi: 10.3390/antiox13040396 (PMC11047703; doi:10.3390/antiox13040396)
Supplement: Supplementary file 1 [file antioxidants-13-00396-s001.zip › antioxidants-2893419-supplementary.pdf]

antioxidants

Supplementary Materials

Article

Endothelial NOX5 Obliterates the Reno-Protective Effect of Nox4 Deletion by Promoting Renal Fibrosis via Activation of EMT and ROS-Sensitive Pathways in Diabetes

Karin A. M. Jandeleit-Dahm [1,2], Haritha R. Kankanamalage [1], Aozhi Dai [1], Jaroslawna Meister [2], Sara Lopez-Trevino [1], Mark E. Cooper [1], Rhian M. Touyz [3], Christopher R. J. Kennedy [4] and Jay C. Jha [1,*]

1 Department of Diabetes, School of Translational Medicine, Monash University, Alfred Medical Research & Education Precinct, Melbourne, VIC 3004, Australia

2 Institute for Clinical Diabetology, German Diabetes Centre, Leibniz Centre for Diabetes Research at Heinrich Heine University, 40225 Düsseldorf, Germany

3 Research Institute of the McGill University Health Centre, McGill University, Montreal, QC H3H 2R9, Canada

4 Department of Medicine, Kidney Research Centre, Ottawa Hospital Research Institute, Ottawa, ON K1Y 4E9, Canada

* Correspondence: jay.jha@monash.edu

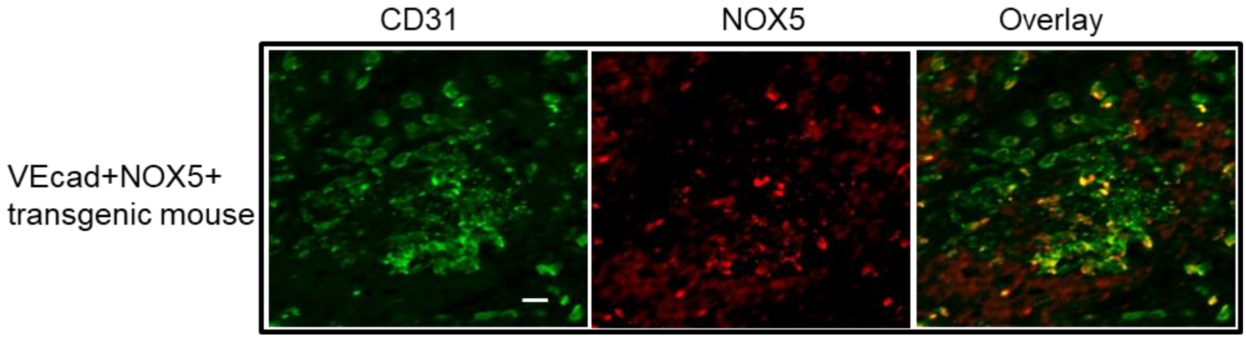

Supplementary Figure S1: NOX5 transgenic mice expressing human NOX5 in endothelial cells (VEcad+NOX5+) in the glomerulus. Co-localisation of CD31 (a marker of endothelial cells; green staining) and NOX5 (red staining) in frozen kidney sections of control VEcad+NOX5+ transgenic mouse. Scale bar, 20 μm in all photomicrographs.

Immunofluorescence: Immunostaining for the co-localisation of NOX5 and CD31 was performed. Double staining for NOX5 and CD31 in mouse frozen kidney sections (5 μm) was performed by incubation with primary antibody to NOX5 (rabbit polyclonal, catalogue no. ab191010; Abcam, Cambridge, MA, USA) and CD31 (goat polyclonal, catalogue no. AF3628; R&D Systems, Minneapolis, MN, USA), followed by incubation with secondary antibody Alexa Fluor 568 (donkey anti-rabbit; Invitrogen, Eugene, OR, USA) and Alexa Fluor 488 (donkey anti-goat; Invitrogen). All stained sections were examined and images were captured using a Nikon eclipse-Ni (Tokyo, Japan) fluorescence microscope.

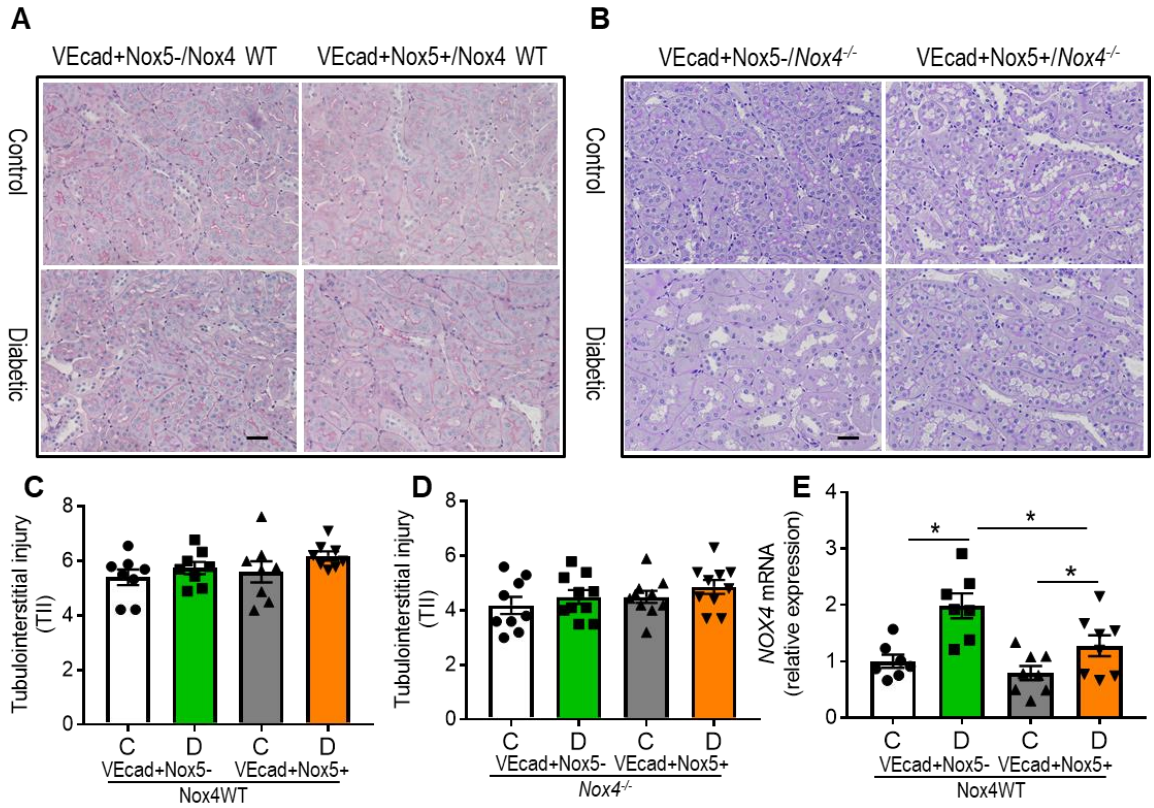

Supplementary Figure S2: Periodic Acid-Schiff staining (**A** and **B**) and the scoring of tubulointerstitial injury, TII (**C** and **D**) in all groups of wild type and Nox4 knockout (*Nox4*$^{-/-}$) mice with and without *NOX5* expression after 10 weeks of STZ- diabetes. Scale bar, 50 μm in all photomicrographs (n = 7–10/gp). Renal cortical gene expression of *Nox4* (**E**) in wild type mice with and without *NOX5* expression after 10 weeks of STZ- diabetes. Data are shown as mean ± SEM. Asterisks represent p-values for comparisons of the indicated groups: * < 0.05.

Supplementary Table S1. Mouse probes and primers for RT-PCR.

Copyright: © 2024 by the authors. Licensee MDPI, Basel, Switzerland. This article is an open access article distributed under the terms and conditions of the Creative Commons Attribution (CC BY) license (https://creativecommons.org/licenses/by/4.0/).

Genes	Gene accession no.	Probe Sequence (6-FAM 5'-3')	Forward Primer 5'-3'	Reverse Primer 5'-3'
Nox2	U43384	CAACTGGACAGGAACCT	AGTGCGTGTTGCTCGACAAG	CCAAGCTACCATCTTATGGAAAGT
Mcp-1	NM_011333	AATGGGTCCAGACATAC	GTCTGTGCTGACCCCAAGAAG	TGGTTCCGATCCAGGTTTTTA
Egr-1	NM_007913	SYBER	TCGGCTCCTTTCCTCACTCA	GCATCATCTCCTCCAGTTTGG
Vimentin	NM_011701	CCGCACCAACGAGA	CGCCATCAACACTGAGTTCAA	TGGCAAAGCGGTCATTCA
Pkc-α	X52685	CGATCCCAGTCCCAG	AGACAAAGACCGGCGACTGT	TTAGCTCTGAGACACCAAAGGAAA
Nox4	NM_015760	CATTTTGCTATTTCATCAAA	AAAAATATCACACACTGAATTCGAGACT	TGGGTCCACAGCAGAAAACTC
Ki-67	NM_001081117	SYBER	CAAAAGGCGAAGTGGAGCTT	TGTTTCGCAACTTTCGTTTGTG
Enos	BC052636	CCAGAGCTACGCACAGC	AACCATTCTGTATGGCTCTGAGACT	CTCTAGGGACACCACATCATACTCAT
α-SMA	NM_007392	TGCCAGATCTTTTCC	GACGCTGAAGTATCCGATAGAACA	GGCCACACGAAGCTCGTTAT
Ctgf	BC006783	ACTGCCTGGTCCAGAC	GCTGCCTACCGACTGGAAGA	CTTAGAACAGGCGCTCCACTCT
Vegf	M95200	CTGTACCTCCACCATGC	GCACTGGACCCTGGCTTTACT	ATGGGACTTCTGCTCTCCTTCTG
Fn1	M10905	CCCCGTCAGGCTTA	ACATGGCTTTAGGCGGACAA	ACATTCGGCAGGTATGGTCTTG
Col4	J04694	CAGTGCCCTAACGGT	GGCGGTACACAGTCAGACCAT	GGAATAGCCGATCCACAGTGA
Tlr4	NM_021297	AAACACCTACCTGGAATGG	GCAGCAGGTGGAATTGTATCG	TGTGCCTCCCCAGAGGATT
Nrf2	NM_001399226	CACAGTGCTCCTATGC	AGCCTCTGTCACCAGCTCAAG	TGTTGTATTTTCACATTGGGATTCA

Disclaimer/Publisher's Note: The statements, opinions and data contained in all publications are solely those of the individual author(s) and contributor(s) and not of MDPI and/or the editor(s). MDPI and/or the editor(s) disclaim responsibility for any injury to people or property resulting from any ideas, methods, instructions or products referred to in the content.
